# Prevalence of Cognitive Impairment/Dementia and Associated Risks Factors Among Community‐Dwelling Elderly People Aged 65 or Above in the Community Centers in Hong Kong: A Cross‐Sectional Study

**DOI:** 10.1002/brb3.70388

**Published:** 2025-03-04

**Authors:** Irelan Tam, Kailu Wang

**Affiliations:** ^1^ The Chinese University of Hong Kong Hong Kong China

## Abstract

**Backgrounds:**

Dementia is a leading cause of morbidity and disability for elderly people worldwide, and prevalence rates of dementia are estimated to be 30% among people ≥80 years old. The burden of the disease could reach 11% of population aged over 60 in 2039.

**Objectives:**

The study aims to report the prevalence rate of cognitive impairment/dementia among community‐dwelling elderly people aged ≥65 years old and identify the associated risk factors for dementia prevention.

**Methods:**

This cross‐sectional study used non‐probability sampling technique. Participants 65 years old were interviewed using a structured questionnaire, and the Cantonese version of Mini‐Mental State Examination was used to screen for cognitive impairment/dementia. CMMSE score of ≤20 was the cut‐off points. Multivariate logistic regression was performed to examine risk factors associated with cognitive impairment/dementia.

**Results:**

From 241 respondents, the study reported the overall prevalence of cognitive impairment/dementia was 18.9%, ranging between 13% and 33% in different age groups. Depression and those who belonged to lower socioeconomic status receiving Comprehensive Social Security Assistance were found to be significant risk factors for cognitive impairment/dementia.

**Conclusion:**

The study showed higher overall prevalence of cognitive impairment/dementia in the community centers than previous studies and potentially high percentage of elderly with undetected dementia in the community. The finding highlighted the importance of increasing dementia literacy, strengthening health promotion for population‐wide screening with follow‐up diagnosis to enable early detection and subsequent interventions and treatments to improve the quality of dementia care for elderly in Hong Kong.

## 1 Background

Dementia is a leading cause of morbidly and disability for elderly people aged ≥65 years old and is one of the key public health issues worldwide including Hong Kong. In 2016, local government reported 9.3% of people aged ≥70 were diagnosed with dementia (Elderly Health Service, Department of Health, Hong Kong 2016). A 2018 meta‐analysis using DSM‐IV/IV‐R criteria reported the prevalence of dementia in Hong Kong was 7.2%, higher than China and Taiwan (Wu et al. 2018). The elderly population ≥65 years old is expected to increase from 21.4% to 31% in 2039 (Healthcare.gov.hk 2022), and the prevalence rates of dementia could be 20%–30% among people aged over 80 (Gov.hk 2023a).

Cognitive impairment/dementia can cause significant memory loss and decline in cognitive domains including comprehension, language, learning capacity, calculation, and judgement (Gov.hk 2023b). Dementia patients were also found to suffer from psychiatric or behavioral problems such as agitation, delusions, anxiety, and aggression (Casey 2015). Research on non‐pharmacological interventions (e.g., sensory simulation, behavior management techniques) suggested that early psychoeducation for caregivers and early recognition of dementia could reduce psychological distress of both patients and their families and could improve caregivers’ mood and quality of life (Lam et al. 2019). Dementia patients could reach 11% of population with aged ≥60 years old in 2039 (Yu 2012).

### 1.1 Theoretical Framework for Cognitive Impairment/Dementia

Cognition (mental processes in learning, solving problems, storage, manipulation, and retrieval of information) is a key to successful health and aging. Cognitive aging theories explain age‐related conditions leading to the decline in basic cognitive abilities (Brown and Park 2003). The theoretical distinction was highlighted between effortful and automatic processes. The elderly with significant decline in effortful processing implied the significant decline in the speed of information processing, working memory, ability to learn and recall new information, and clarity and efficiency of reasoning process as fewer cognitive resources are available for processing, which reflects deficits in memory and cognition.

**FIGURE 1:**
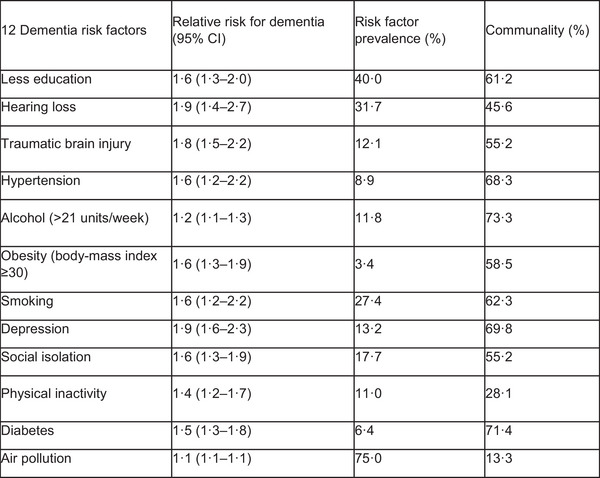
Twelve potentially modifiable risk factors in preventing dementia (Livingston et al. 2020).

Research in cognitive aging seeks to identify and understand the risk factors and distinguish risk factors in correlating to better late‐life cognitive function and late‐life dementia (Tucker‐Drob 2019). Many risk factors have been associated with dementia prevention efforts that include both modifiable (e.g., exposures, lifestyle, and social habits) and nonmodifiable (e.g., age, sex, genetics) risk factors with 12 modifiable risk factors explaining up to 40% of the attributable risk (Figure 1).

### 1.2 Modifiable Risk Factors and the Prevention of Dementia

Modifiable risk was based on the early and midlife risk factors identified in the 2020 Lancet Commission on dementia prevention, intervention, and care. Evidence from life‐course conceptual framework found that smoking, depression, social isolation, physical inactivity, diabetes, and air pollution were key risk factors influencing higher risk for dementia for people aged ≥65 years old (Livingston et al. 2020). Family history or genes and brain injury were found to be major causes of dementia (Mukadam et al. 2019). Other factors included vascular diseases, medications, physical inactivity, and high alcohol consumption (Livingston et al. 2020; Magierski et al. 2020).

People with uncontrolled vascular risk factors, which associate with low education levels, are found to be more vulnerable to cognitive decline (Lam et al. 2019). By modifying lifestyle and improving brain health could reduce the risks of cognitive impairment/dementia (Livingston et al. 2020). The 2017 Lancet Commission identified nine modifiable risk factors, and 39.5%, 41.2%, and 55.8% of proportion of dementia cases could be theoretically preventable in China, India, and six Latin American countries, respectively (Mukadam et al. 2019). The finding provided insights that improving education, management of high blood pressure, treatment of hearing loss, reducing physical inactivity, controlling smoking, reducing alcohol consumption, reducing social isolation, management of socio‐economic, and commercial drivers of obesity and depression could reduce the risks of cognitive impairment/dementia (Dukelow et al. 2023).

### 1.3 Significance of the Study

According to the World Health organization, dementia has physical, psychological, social, and economic impacts on people with dementia, bringing heavy financial burden to their carers, families, and society as well (Who.org 2023). The aging population is becoming a major concern in Hong Kong as population ≥65 years old will reach 31% in 2039 (Gov.hk 2023c). There were limited studies on the prevalence of dementia in Hong Kong. A 2008 comprehensive study reported 17.4% for people aged ≥70 years old (Lam et al. 2018), and two previous studies used in a systematic review showed higher prevalence in Hong Kong over 16 years (Chiu et al., 1998 and Lam et al., 2008).

There were fewer dementia studies among community‐dwelling elderly in community centers in the Hong Kong context. A 2019 systematic review on global cognitive impairment prevalence of elderly >50 years old living in the community reported prevalence ranged between 5.1% and 41% with a median of 19.0% (Pais et al. 2020). Research highlighting the barriers to diagnosis and care were mainly attributed to the lack of awareness and understanding of dementia, and even high‐income countries with advanced healthcare systems showed rather low diagnosis rate (Connolly et al. 2011). A study in China showed undetected dementia of 77.5% and 93.5% in urban and rural residents, respectively (Qi et al. 2020). Due to potentially high undetected dementia in the community centers, there was a need to estimate the level of dementia prevalence in the community setting beyond doctor diagnosis.

There were also fewer dementia studies evaluating the modifiable risk factors that associated with their lifestyle in the Hong Kong context as most studies on risk factors of dementia/mild cognitive impairment were commonly conducted in the clinical setting (Xu et al. 2020). Fewer published studies were conducted in evaluating the risk of lifestyle and vascular risk factors in association with cognitive impairment/dementia in the community centers. Therefore, study on identifying relevant modifiable risk factors can enable better control of the conditions and timely access to care and support (Prince et al. 2011). The current study could contribute to dementia research by reporting the latest prevalence of cognitive impairment/dementia and assisting the predication of dementia at the population level. By identifying relevant modifiable risks, the outcome of the study could drive discussion on public health strategies in tailoring prevention and/or intervention programs for the population with a higher risk of dementia.

### 1.4 Research Objectives

Based on the research gaps identified above, the study aims to determine the prevalence of cognitive impairment/dementia and its associated risks factors using Cantonese version of Mini‐Mental State Examination (CMMSE) as the cognitive scanning tool among community‐dwelling elderly people aged ≥65 years old in Hong Kong community centers.

Also, it is hypothesized that the nine modifiable risk factors, namely lower education level, hypertension, hearing loss, physical inactivity, smoking, excessive alcohol consumption, social isolation, depression, and diabetes are associated with higher risks of cognitive impairment/dementia.

## 2 Methods

### 2.1 Study Design and Procedure

This cross‐sectional study was conducted in the period between December 2023 and March 2024 to estimate the prevalence rate of cognitive impairment/dementia. The study has been approved by the ethics committees of the Chinese University of Hong Kong, and verbal consent was obtained from all respondents before the interview. For respondents who have moderate‐to‐severe dementia and are unable to give consent, their relatives were consulted to give consent before the interview.

### 2.2 Study Sample and Data Collection

**FIGURE 2:**
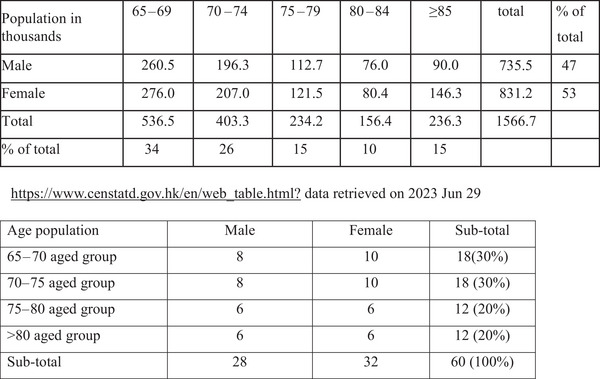
Quota sample size per a community center based on sex and aged group.

The inclusion criteria for participants are aged ≥65 years old in the community centers and are residents in Hong Kong. The study adopted quota sampling approach as random sampling may not be achievable due to resource constraints (Figure 2). Sample size was computed to be 307 based on the expected prevalence of 20% and expected response rates of 80% with a confidence level of 95%. All respondents were interviewed by either the researcher or trained volunteers for administering the diagnosis questionnaire.

### 2.3 Study Instruments and Measurements

The questionnaire that included nine risk factors based on local and/or validated measures is shown in Figure 3.

FIGURE 3Measures to be used in the study.

**Figure:**
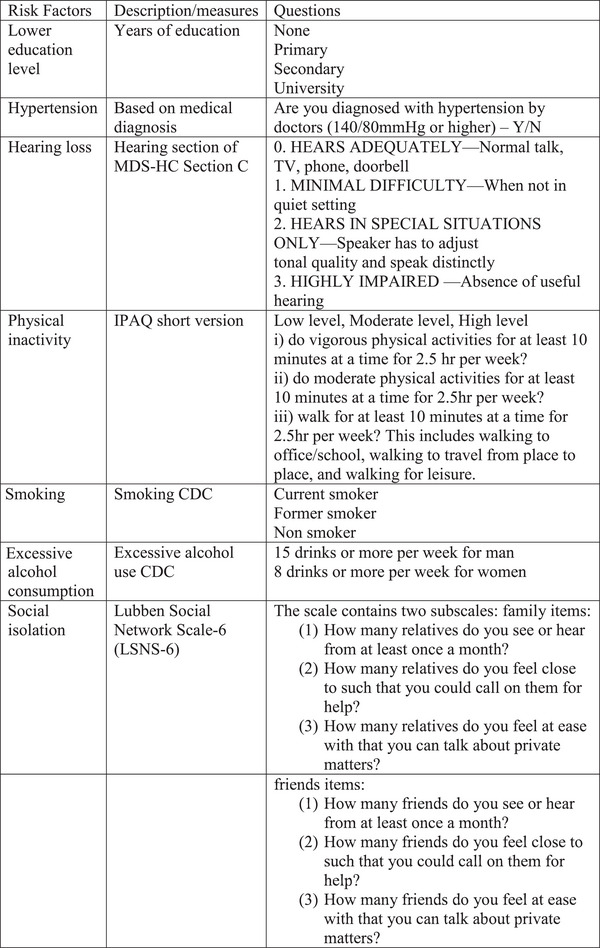

**Figure:**
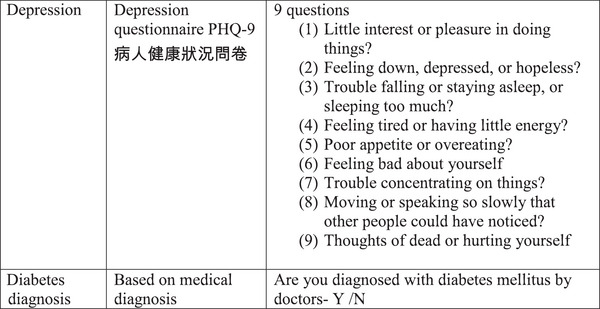

Mini‐Mental State Examination (MMSE) is a diagnostic test for dementia and is used extensively in clinical, research, and community settings for screening patients with or without cognitive impairments/dementia. The current study used the Cantonese version of Mini‐Mental State Examination (CMMSE), which is a validated instrument as a screening tool in clinical and research, and Cronbach’ *α* and test‐retest reliability were 0.86 and 0.78, respectively (Chiu et al. 1994). The CMMSE cut‐off scores of ≤20, with sensitivity and specificity of 97.5% and 97.3%, respectively, were defined as cognitive impairment/dementia (Katzman et al. 1988). A pilot study of ∼10 people aged ≥65 years old in one community center was conducted to assess the validity and reliability of the questionnaires before thisstudy. The Cronbach’ *α* of the current study was 0.58. The small Cronbach's *α* could be due to small sample size.

Cantonese version of Lubben Social Network Scale (LSNS6) was used for screening social isolation, and the cut‐off points of 12 were defined as at risk for social isolation (Lubben et al. 2006). The internal consistency of LSNS6 reported Cronbach’ *α* as 0.75 (Chan et al. 2023). The Cronbach’ *α* of the current study was 0.83.

Cantonese version of Patient Health Questionnaire (PHQ9) was used for screening depression. The cut‐off points of 0–4 indicates no depressive symptoms, 5–9 mild depressive symptoms, 10–14 moderate depressive symptoms, 15–19 moderately‐severe depressive symptoms, and 20–27 severe depressive symptoms. The internal consistency of PHQ9 reported Cronbach’ *α* as 0.82 (Yu et al. 2012). The Cronbach’ *α* of the current study was 0.83.

The Cantonese version of the International Physical Activity Questionnaire (IPAQ‐SF) was used for self‐reported physical activity. The instrument reported intra‐class correlation for total physical activity was 0.75 (Macfarlane et al. 2007). The metabolic equivalent (MET) minutes per week was used, <495 MET‐mins/week indicates inactive, >495 and <3000 MET‐mins/week indicates moderate‐intensity activity, and >3000 MET‐mins/week indicates high‐intensity activity. The Cronbach’ *α* of the current study was 0.13. The low Cronbach’ *α* may be due to self‐report bias.

### 2.4 Statistical Analysis

The collected data were analyzed using IBM SPSS Statistics version 29. Prevalence was computed based on the proportion of respondents who had a CMMSE score ≤20 out of the total number of respondents screening for cognitive impairment/dementia. A descriptive analysis was carried out, and continuous variables were expressed as means ± SD. Nine risk factors were reported as categorical variables and were summarized as frequencies and percentages. Multiple logistic regression analysis was used to identify factors associated with cognitive impairment/dementia. Independent variables, which had VIF >6 were excluded in the regression analysis, unstandardized regression coefficients (β) and odds ratios (ORs), and their 95% confidence intervals (CIs) were used to evaluate the associations between variables, and statistically significant differences were considered when *p* < 0.05.

## 3 Results

**FIGURE 4:**
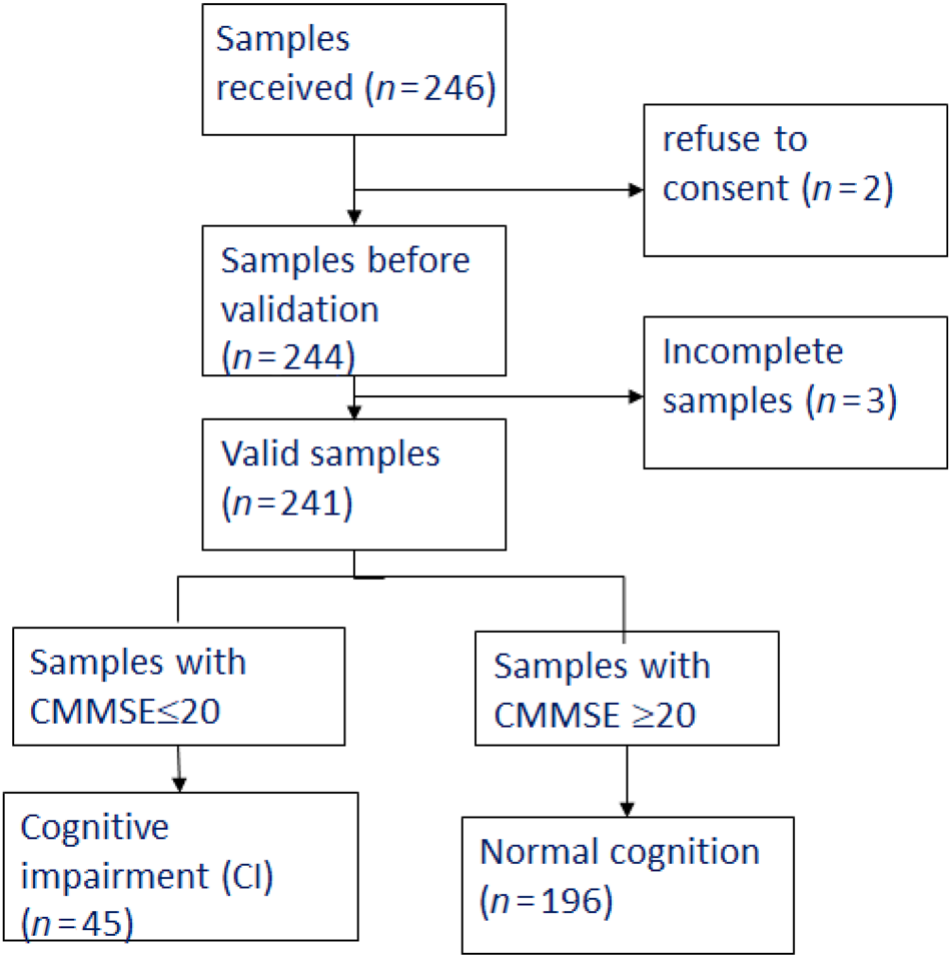
Two hundred forty‐one valid samples used in the study.

In total, 246 questionnaires were collected, three of them did not report CMMSE data and two respondents refused to answer, and 241 valid samples were used (Figure 4). The study included 241 participants, of whom 39% were males and 61% were females. Note that 43% of the sample attained secondary or above secondary education, whereas 57% had below primary education. The median age was 71–75 category, and 49% of respondents lived in Tsuen Wan and Kwai Tsing area. The mean CMMSE was 22.8 ±2.6, ranging 14–30. Using CMMSE ≤20 cut‐offs, the prevalence of CI was 18.7%. The demographic data are shown in Table 1. A higher prevalence of CI was found in those with age ≥80 years old (*n* = 13, 33.3%) and female (*n* = 30, 20.4%). The prevalence of dementia by age group and gender is shown in Table 2.

**TABLE 1:** Demographic data of the study (*n* = 241).

	Total (*n* = 241)	Normal (CMMSE ≥ 20) (*n* = 196)	Impaired (CMMSE ≤ 20) (*n* = 45)	Statistic	*p*
CMMSE mean	22.8 ± 2.6	23.7 ± 1.9	18.9 ± 1.4	*F* (1239) = 239.67	<0.001
Age 65–70 71–75 76–80 >80	84 (34.9%) 67 (27.8%) 51 (21.2%) 39 (16.2%)	69 (35.2%) 58 (29.6%) 43 (21.9%) 26 (13.3%)	15 (33.3%) 9 (20.0%) 8 (17.8%) 13 (28.9%)	*χ* ^2^ (3) = 7.068	0.07
Gender Male (%) Female (%)	94 (39.0%) 147 (61.0%)	79 (40.3%) 117 (59.7%)	15 (33.3%) 30 (66.7%)	*χ* ^2^ (1) = 0.748	0.39
Education Primary and below Secondary and above	138 (57.3%) 103 (42.7%)	107 (54.6%) 89 (45.4%)	31 (68.9%) 14 (31.1%)	*χ* ^2^ (1) = 3.057	0.10
District HK Kln NT	10 (4.1%) 71 (29.5%) 160 (66.4%)	6 (3.1%) 59 (30.1%) 131 (66.8%)	4 (8.9%) 12 (26.7%) 29 (64.4%)	*χ* ^2^ (2) = 3.174	0.21

**TABLE 2:** Prevalence of cognitive impairment/dementia by age group and by gender (*n* = 241).

Prevalence rate by subgroup	Total (*n* = 241)	Normal (CMMSE ≥ 20) (*n* = 196)	Impaired (CMMSE ≤ 20) (*n* = 45)
Overall	241 (100%)	196 (81.3%)	45 (18.7%)
Age 65–70 71–75 76–80 >80	84 (100%) 67 (100%) 51 (100%) 39 (100%)	69 (82.1%) 58 (86.6%) 43 (843%) 26 (667%)	15 (17.9%) 9 (13.4%) 8 (15.7%) 13 (33.3%)
Gender Male Female	94 (100%) 147 (100%)	79 (84.0%) 117 (79.6%)	15 (16.0%) 30 (20.4%)
Education Primary and below Secondary and above	138 (100%) 103 (100%)	107 (77.5%) 89 (86.4%)	31 (22.5%) 14 (13.6%)

Higher prevalence for elderly with moderate‐to‐severe depression (*n* = 16, 42.1%), among those with lower education levels (*n* = 31, 22.5%), among those with limited social network (*n* = 26, 19.5%), among those physically inactive elderly (*n* = 39, 19.5%), among those with mild to impaired hearing (*n* = 20, 22.5%) and those receiving Comprehensive Social Security Assistance (CSSA) (*n* = 16, 31.4%). The prevalence of risk factors is shown in Table 3.

**TABLE 3:** Prevalence of cognitive impairment/dementia by risk factors (*n* = 241).

	Total (*n* = 241)	Normal (CMMSE ≥ 20) (*n* = 196)	Impaired (CMMSE ≤ 20) (*n* = 45)	Prevalence Rates (%)	Statistic	*p*
Hearing—MDS Normal Mild to impaired	152 (63.1%) 89 (36.9%)	127 (64.8%) 69 (35.2%)	25 (55.6%) 20 (44.4%)	16.4 22.5	*χ* ^2^ (1) = 1.342	0.30
Social Isolation LSNS6 <12 isolation >12 normal	133 (55.2%) 108 (44.8%)	107 (54.6%) 89 (45.4%)	26 (57.8%) 19 (42.2%)	19.5 17.6	*χ* ^2^ (1) = 0.150	0.74
LSNS6 mean	13 ± 7	13 ± 7	13 ± 8		*F* (1,239)=0.008	0.93
Physical inactivity IPAQ Inactive moderate	200 (83.0%) 41 (17.0%)	161 (82.1%) 35 (17.9%)	39 (86.7%) 6 (13.3%)	19.5 14.6	*χ* ^2^ (1) = 0.53	0.66
MET mean^a^	482 ± 388	495 ± 402	432 ± 322		*F*(1,239)=0.95	0.33
Depression PHQ9 Mild and below Moderate to severe	203 (84.2%) 38 (15.8%)	174 (88.8%) 22 (11.2%)	29 (64.4%) 16 (35.6%)	14.3 42.1	*χ* ^2^ (1) = 16.313	<0.001^*^
PHQ9 Mean	5 ± 5	5 ± 5	7 ± 6		F(1,239)=7.977	0.005
Hypertension Normal with hypertension	125 (51.9%) 116 (48.1%)	101 (51.5%) 95 (48.5%)	30 (66.7%) 15 (33.3%)	24.0 12.9	*χ* ^2^ (1) = 4.854	0.03^*^
CVD Normal with CVD	182 (75.5%) 59 (24.5%)	148 (75.5%) 48 (24.5%)	34 (75.6%) 11 (24.4%)	18.7 18.6	0 Cells have expected count less than 5	
DM Normal with DM	189 (78.4%) 52 (21.6%)	152 (77.6%) 44 (22.4%)	37 (82.2%) 8 (17.8%)	19.6 15.4	*χ* ^2^ (1) = 0.472	0.55
Hereditary No Yes	165 (68.5%) 76 (31.5%)	132 (67.3%) 64 (32.7%)	33 (73.3%) 12 (26.7%)	20.0 15.8	*χ* ^2^ (1) = 0.607	0.48
CSSA No Yes	189 (78.8%) 51 (21.2%)	160 (82.1%) 35 (17.9%)	29 (64.4%) 16 (35.6%)	15.3 31.4	*χ* ^2^ (1) = 6.773	0.01^*^
Smoking Non‐smoker smoker	232 (96.3%) 9 (3.7%)	188 (95.9%) 8 (4.1%)	44 (97.8%) 1 (2.2%)	19.0 11.1	*χ* ^2^ (1) = 0.352	0.55
Drink Non‐drinker Moderate to heavy	235 (97.5%) 6 (2.5%)	190 (96.9%) 6 (3.1%)	45 (100.0%) 0	19.1 0.0	*χ* ^2^ (1) = 1.413	0.60

^a^ MET minutes represent the amount of energy expended carrying out physical activity.

^*^ *p* < 0.05

Using multivariate logistic regression, depression (OR = 5.816, CI = 2.060–13.058, *p* < 0.001), hypertension (OR = 0.407, CI = 0.169–0.981, *p* = 0.05), and who belonged to a lower socioeconomic status receiving CSSA (OR = 2.641, CI = 1.149–6.073, *p* = 0.02) were found to be significant risk factors for cognitive impairment/dementia. Other factors including age group >80 (OR = 2.389, CI = 0.821–6.951, *p* = 0.11), cardiovascular disease (OR = 2.388, CI = 0.728–7.828, *p* = 0.15), and female sex (OR = 1.546, CI = 0.709–3.372, *p* = 0.273) were associated with higher risk of cognitive impairment/dementia; however, they were found to be insignificant in the sample (Table 4).

**TABLE 4:** Logistic regression analyzes risk factors for cognitive impairment/dementia.

Independent variables	Model 1		Model 2			Model 3			Model 4	
	OR	95% CI	OR	95% CI		OR	95% CI	OR		95% CI
Gender female	1.480	0.734–2.982	1.339	0.674–2.868	1.430		0.672–3.043	1.546		0.709–3.372
Age group 71–75 Age group 76–80 Age group >80	0.716 0.884 2.443^*^	0.292–1.760 0.344–2.268 1.014–5.887	0.634 0.682 2.438	0.248–1.622 0.241–1.927 0.947–6.276	0.671 0.684 2.445		0.255–1.771 0.234–2.000 0.885–6.757	0.641 0.581 2.389		0.235–1.743 0.189–1.791 0.821–6.951
District Kln District NT			0.265 0.214^*^	0.059–1.18 0.051–0.891	0.171^*^ 0.138^*^		0.035–0.836 0.030–0.648	0.184^*^ 0.159^*^		0.036–0.941 0.032–0.782
Education: Above primary			0.563	0.266–1.194	0.576		0.262–1.265	0.572		0.253–1.293
Receiving CSSA			2.693^*^	1.245–5.824	2.712^*^		1.201–6.123	2.641*		1.149–6.073
MDS: Mild to impaired					1.177		0.542–2.559	1.216		0.553–2.671
IPAQ: Moderate					1.120		0.393–3.197	1.058		0.356–2.146
PHQ9: Moderate severe					5.544^*^		2.286–13.444	5.186^*^		2.060–13.058
LSNS6: Social isolation					0.727		0.330–1.600	0.797		0.357–1.784
Hypertension								0.407^*^		0.169–0.981
Cardiovascular disease								2.388		0.728–7.828
Hereditary								0.893		0.297–2.680

* *p* < 0.05.

## 4 Discussion

The current study aimed to find the prevalence rate of cognitive impairment/dementia of elderly people aged ≥65 years old in the community centers and evaluate the nine risk factors that were associated with the risks of cognitive impairment/dementia. CMMSE ≤20 was used as cut‐off points. The current study reported the overall prevalence of cognitive impairment/dementia to be 18.7%, which is higher than two previous Hong Kong studies that reported prevalence rates of 9.3% in 2019 (Lam et al. 2019) and 14% in 2016 (Shea et al. 2016). The prevalence rate of cognitive impairment/dementia ranged from 13.4% to 33.3%, and the highest prevalence was observed among elderly aged >80 years old, which was in line with 2012 research finding of 20%–30% (Yu 2012) and 2013 baseline study of 33% (C. H. Wong et al. 2013). Also, higher prevalence was observed among females than male (20.4% vs. 16.0%) and among those with higher education levels compared to those with lower education levels (22.5% vs. 13.6%), which were in line with the 2016 study (Shea et al. 2016). Higher prevalence was observed among those who belonged to a lower socioeconomic status receiving CSSA compared to those who belonged to a higher socioeconomic status group (31.4% vs. 15.3%), elucidating poor social and economic conditions could increase the risks of dementia.

During the interview with respondents, most of them had rather low dementia literacy. The limited knowledge and low awareness may be the reason for the underdiagnosis of cognitive impairment/dementia in the community. Some respondents were worried after the interview and reported that they were unable to have follow‐up cognitive evaluation and assessment due to long waiting time for appointment booking on diagnostic services at public hospitals (Heung 2024).

The logistic regression results showed two risk factors, depression and those who belonged to lower socioeconomic status receiving CSSA, were associated with higher risk of cognitive impairment/dementia. Note that 15.8% of the respondents (*n* = 38) over aged ≥65 years old reported to have moderate‐to‐severe depression symptoms, which was higher than 8.6% of CUHK 2023 mental health survey (CUHK.edu.hk 2023). It is crucial to address late‐life depression in order to reduce the risk of cognitive impairment/dementia. Note that 21.2% of respondents (*n* = 51), who received CSSA belonged to poor socioeconomic status and lower education levels, were found to be a significant risk factor. Research showed people with low socioeconomic status and lower education levels were experiencing poor physical and mental health and were associated with higher prevalence of cognitive impairment/dementia (Zhang et al. 2022). Also, 55% of respondents (*n* = 133) were found to have extremely limited social network and high risk for isolation, who may have health problems and need intervention for better health and functioning in the community (E. L. Wong et al. 2023).

Studies indicated that social isolation and inactivity were associated with higher risk of dementia (Lang et al. 2017); however, they were found to be insignificant in the sample. The possible barrier to diagnosis could be attributable to the fact that dementia could be a stigma in Chinese culture and elderly people may feel uncomfortable to share with others. There may be a cause of underdiagnosis of dementia in the community.

Risk factors including hearing loss, depression, physical inactivity, low social contact, and diabetes reported higher prevalence than a previous cross‐sectional regional study (Mukadam et al. 2019) (Table 5).

**TABLE 5:** Risk factors of cognitive impairment/dementia compared with (Mukadam et al. 2019).

	RR (95% CI) for dementia in China (*n* = 2162) (Mukadam et al. 2019)	Risk factor prevalence (%)	Current study: Adjusted OR (95% CI) for cognitive impairment/dementia (*n* = 241)	% of sample with cognitive impairment/dementia (*n* = 45)
Low education	1.6 (1.3–2.0)	76	Below primary 0.754 (0.336–1.692)	31
Hearing loss	1.9 (1.4–2.7)	14	1.290 (0.581–2.865)	44
Hypertension	1.6 (1.2–2.2)	38	0.408* (0.169–0.982)	33
Smoking	1.6 (1.2–2.2)	23	1.302 (0.121–14.047)	2
Depression	1.9 (1.6–2.3)	2	5.539* (2.218–13.833)	36
Physical inactivity	1.4 (1.2–1.7)	51	0.956 (0.316–2.895)	87
Low social contact	1.6 (2.3–1.9)	3	0.797 (0.357–1.784)	58
Diabetes mellitus	1.5 (1.3–1.8)	9	0.771 (0.076–7.859)	18

### 4.1 Implications for Public Health

The cross‐sectional study reported higher overall prevalence rates of cognitive impairment/dementia for elderly ≥65 years old than previous studies in Hong Kong. With the aging population, the high prevalence in the age group >80 years old implied the heavy financial burden on the government and society. As dementia would be one of the major contributors to disability and have exerted high burdens on caregivers as well as health and social care systems (Lam et al. 2019), government should implement community‐based services and long‐term care.

The study highlighted the importance of raising awareness/screening of cognitive impairment/dementia in the community as many elderly people with dementia were either undiagnosed or unaware of the diagnosis and lack knowledge about modifiable risks factors for dementia. A systematic review and meta‐analysis found >60% undetected dementia in the community setting (Lang et al. 2017). There is a need to strengthen health promotion for population‐wide screening with follow‐up diagnosis to enable early detection and subsequent intervention to improve the quality of dementia care. Research found that early diagnosis would allow elderly people to access better medical and community care such as outpatient and inpatient treatment and counselling (Prince et al. 2011).

The risk factors analysis revealed the importance of intervention to reduce depression and hypertension risk in order to reduce risk of developing cognitive impairment/dementia. Additionally, government should allocate sufficient resources to help underprivileged elderly people to access diagnosis, follow‐up treatment, and obtain continuous community support service to improve their health conditions.

### 4.2 Limitation and Future Study

There were several limitations in the current study that have affected the result. First, the challenge of time constraints and small sample size limit the statistic power and generalizability. Second, it was difficult to recruit respondents in the elderly home as many centers refused the invitation to conduct research during the high seasonal influenza period. Third, there may be selection bias as random sampling was not possible. Fourth, there were self‐report bias as respondents may make socially acceptable answers rather than being truthful, and several potential sources of bias should be considered in future studies. Fifth, despite training was provided to the volunteers supporting the study, they had diverse skill and competencies and may impact the data quality in the research study. Sixth, a cross‐sectional study design cannot establish causal relationships between the risk factors and cognitive impairment/dementia, and further longitudinal studies could provide more insights into these associations. Finally, although the CMMSE has been proven to be a reliable instrument for the screening of cognitive impairment/dementia, other methods of assessment such as computed tomography scans, magnetic resonance imaging, and blood tests could support a definitive diagnosis.

Further research should increase the sample size and train more volunteers and healthcare workers to support community screening, strengthen data accuracy, and use objective measures for lifestyle factors to improve the quality of statistical analysis in cognitive impairment/dementia's study. Future longitudinal study examining the cognitive trajectory trend of modifiable risk factors in the population can provide deeper insights into dementia care and disease prevention.

## 5 Conclusion

In conclusion, cognitive impairment/dementia is a significant health and social problem in Hong Kong. The study found the overall prevalence of cognitive impairment/dementia was 18.9% ranging between 13% and 33% in different age groups. Depression and those who belonged to lower socioeconomic status receiving CSSA were found to be significant risk factors. Potentially a high percentage of elderly people with dementia may be either undiagnosed or unaware of the disease. The outcomes of the study highlighted the importance of increasing awareness/screening of cognitive impairment/dementia and identifying respective risk factors is critical in developing public health strategies for dementia prevention in the community. The finding also suggested a need to strengthen health promotion for population‐wide screening and follow‐up diagnosis for elderly to enable early detection of cognitive impairment/dementia and subsequent interventions and treatments to improve the quality of dementia care for elderly in Hong Kong.

## Author Contributions

**Irelan Tam**: conceptualization, methodology, writing–review and editing, software, writing–original draft, formal analysis, project administration, data curation. **Kailu Wang**: Supervision, resources.

### Peer Review

The peer review history for this article is available at https://publons.com/publon/10.1002/brb3.70388

## Data Availability

The data that support the findings of this study are available from the corresponding author upon reasonable request.
